# pyrazinecarboxamides as Potential Elicitors of Flavonolignan and Flavonoid Production in *Silybum marianum* and *Ononis arvensis* Cultures *In Vitro*

**DOI:** 10.3390/molecules16119142

**Published:** 2011-11-01

**Authors:** Lenka Tumova, Jiri Tuma, Martin Dolezal

**Affiliations:** 1 Department of Pharmacognosy, Faculty of Pharmacy in Hradec Kralove, Charles University in Prague, Heyrovskeho 1203, 500 05 Hradec Kralove, Czech Republic; 2 Department of Biology, Faculty of Science, University of Hradec Kralove, Rokitanskeho 62, 500 03 Hradec Kralove, Czech Republic; 3 Department of Pharmaceutical Chemistry and Pharmaceutical Analysis, Faculty of Pharmacy in Hradec Kralove, Charles University in Prague, Heyrovskeho 1203, 500 05 Hradec Kralove, Czech Republic

**Keywords:** abiotic elicitors, amides of pyrazinecarboxylic acids, flavonoid, flavonolignan, *Silybum marianum*, *Ononis arvensis*

## Abstract

The effect of new synthetic pyrazinecarboxamide derivatives as potential elicitors of flavonolignan and flavonoid production in *Silybum marianum* and *Ononis arvensis* cultures *in vitro* was investigated. Both tested elicitors increased the production of flavonolignans in *S. marianum* callus and suspension cultures and flavonoids in *O. arvensis* callus and suspension cultures. Compound **I**, 5-(2-hydroxybenzoyl)-pyrazine-2-carboxamide, has shown to be an effective elicitor of flavonolignans and taxifoline production in *Silybum marianum *culture *in vitro.* The maximum content of silydianin (0.11%) in *S. marianum* suspension culture was induced by 24 h elicitor application in concentration of 1.159 × 10^−3^ mol/L. The maximum content of silymarin complex (0.08%) in callus culture of *S. marianum* was induced by 168 h elicitor application of a concentration 1.159 × 10^−4^ mol/L, which represents contents of silydianin (0.03%), silychristin (0.01%) and isosilybin A (0.04%) compared with control. All three tested concentrations of compound **II**, *N*-(2-bromo-3-methylphenyl)-5-*tert*-butylpyrazin-2-carboxamide increased the flavonoid production in callus culture of *O. arvensis* in a statistically significant way. The best elicitation effect of all elicitor concentrations had the weakest c_3_ concentration (8.36 × 10^−6^ mol/L) after 168 h time of duration. The maximum content of flavonoids (about 5,900%) in suspension culture of *O. arvensis* was induced by 48 h application of c_3_ concentration (8.36 × 10^−6^ mol/L).

## 1. Introduction

*In vitro* cultures have been seen as an alternative source of biologically active compounds [1]. The disadvantage of these cultures is low production of secondary metabolites, therefore new methods for higher production and accumulation of secondary metabolites by cultures *in vitro* are being constantly evaluated. One of these methods is the method of elicitation.

The elicitor can be regarded as a stress factor involved in the plant-microorganism, plant-pesticide, plant heavy metal or plant-UV irradiation reactions. Due to chemical defensive reactions, signal substances (elicitor) increase the activity of certain enzymatic systems for a short period and these systems catalyse the formation of stress substances similar to the particular secondary metabolites [2].

Our previous studies were focused on derivatives of pyrazinecarboxylic acids as elicitors. These pyrazine derivatives were originally prepared as antimycobacterial or antifungal compounds at the Department of Pharmaceutical Chemistry and Pharmaceutical Analysis, Faculty of Pharmacy in Hradec Králove. Interesting *in vitro* antimycobacterial activity was found for *N*-(3-iodo-4-methylphenyl)pyrazine-2-carboxamide (MIC < 2.0 μmol/L against *M. tuberculosis*) and 5-*tert*-butyl-6-chloro-*N*-(3-iodo-4-methyl-phenyl)pyrazine-2-carboxamide [3]. These derivatives also inhibit photosynthesis in spinach chloroplasts [4].

**Figure 1:**
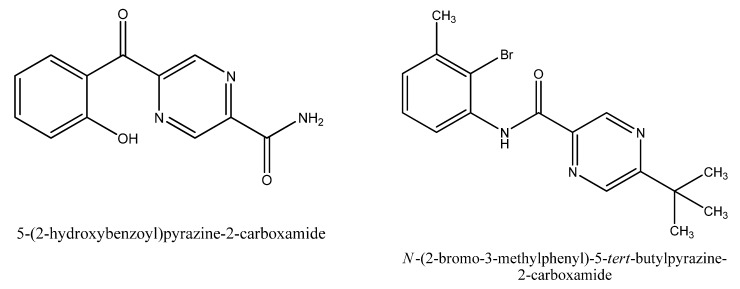
Compounds **I** and **II**.

Substituted pyrazinecarboxamides markedly influenced production of flavonolignans in *Silybum marianum *callus and suspension cultures, particularly two compounds: *N*-(3-iodo-4-methylphenyl)pyrazine-2-carboxamide and *N*-(3-iodo-4-methylphenyl)-5-*tert-*butyl-pyrazine-2-carboxamide [5]. The higher production of flavonolignans in *S. marianum* callus and suspension cultures were found when as elicitors 5-*tert*-butyl-*N*-*m*-tolylpyrazine-2-carboxamide and *N*-(5-bromo-2-hydroxyphenyl)-5-*tert*-butyl-6-chlorpyrazine-2-carboxamide have been used [6]. Homolytic aroylation of pyrazine nucleus with various substituted aromatic carbohydrates afforded a series of 5-aroylpyrazine-2-carboxylic acid derivatives [7]. Among the sixteen derivatives prepared, 5-(2-hydroxy-benzoyl)-pyrazine-2-carboxamide (compound **I**, Figure 1) was chosen for evaluation as potential elicitors of flavonolignan and flavonoid production in *S. marianum* and *O. arvensis* cultures *in vitro.*

The objective of this study was to verify other derivatives as elicitors on flavonoid production in callus and suspension culture of *Ononis arvensis* and on separately substances of silymarin complex in *Silybum marianum *culture *in vitro.*

Restharrow root from *Ononis spinosa* or *Ononis arvensis* L. (Fabaceae) is used as a mild diuretic. The action of the drug has been confirmed in animal experiments, but so far it has not been possible to isolate any active constituents nor have any of its constituents of known structure been tested pharmacologically. In folk medicine restharrow root is also used for gout and rheumatism complaints.

The drug contains 0.02–0.1% essential oil, with *trans*-anetole as the main component, along with carvone and menthol, isoflavones, especially ononin (formononetin 7-glucoside) and its 6′′-malonate, and also biochanin A 7-glucoside and its 6′′-malonate, triterpenes, especially α-onocerin (onocol), and sterols, particularly sitosterol [8].

Silymarin from milk thistle *(Silybum marianum)* is a phytomedicine used for prophylaxis and treatment of liver damage caused by metabolic toxins (alcohol, tissue poisons), after hepatitis, liver cirrhosis and fatty liver. The extract of *S. marianum*—silymarin is the mixture of flavanolignans, namely silybin, silydianin and silychristin. Most of hepatoprotective properties are attributed to silybin. It has also been found that silybin increases the rate of synthesis of ribosomal nucleic acids through stimulation of the nucleolar polymerase I. This reinforces protein synthesis and accelerates cell-regeneration processes, so that, besides the prophylactic action, there may also be a curative effect. Other components are the 3-desoxy-derivatives of silychristin and silydianin, isosilychristin, isosilybinin and its 3 desoxyderivative silandrin, the 3-desoxycompounds silyhermin, neosilyhermin A and B, 2,3-dehydrosilybin, as well as tri- to pentamers of silybin; other constituents are taxifolin, quercetin, dihydrokaempferol, kaempferol, apigenin, naringenin, eriodyction, chrysoeriol, and 5,7-dihydroxychromone; c.a. 20–30% fixed oil with a high proportion of linoleic acid; sterols-cholesterol, stigmasterol, campesterol, sitosterol [9].

Compounds of the silymarin complex have also other interesting activities, such as anticancer and cancer protective and hypocholesterolemic properties. Proapoptotic activity of silybin in pre-and/or cancerogenic cells and anti-angiogenic activity of silybin show other important activities that bring silymarin preparations closer application in the cancer treatment [10]. Compounds of the silymarin complex show also antioxidant activity [11].

## 2. Results and Discussion

Successful elicitation is subject to many factors that are specific for each elicitor and for each explant culture. This work focused on the elicitor types, elicitor concentration and time duration of elicitor´s effect. The results (Table 1 and Table 2) indicate that the elicitor used during the production of flavonolignans in *S. marianum* callus and suspension cultures influenced the production favourably.

**Table 1:** Content of silymarin complex substances (%) and taxifolin (%) in *Silybum marianum* callus culture afer elicitor treatment.

Compound I (conc. mol/L)	Time of sampling (hours)	TAX (%)	SILYD (%)	SILYCHR (%)	ISOSILYB A (%)	ISOSILYB B (%)	SILYB A (%)	SILYB B (%)	SILYM COMP (%)
**C_1_ = 1.159 × 10^−3^**	control	0	-	0	-	0	-	-	0
	6	0	-	-	0	-	0	-	0
	12	0	0.04	0.01	-	-	-	-	0.05
	24	0	0.04	0	-	-	-	-	0.04
	48	0	-	0	0	0	-	-	0
	72	0	-	-	0	0	-	0	0
	168	0	0	0	0	0	0	-	0
**C_2_ = 1.159 × 10^−4^**	control	0	-	0.01	-	-	-	-	0.01
	6	0	-	0.01	-	-	-	-	0.01
	12	0	-	0.01	-	0	-	-	0.01
	24	0	0.03	0	-	-	-	-	0.03
	48	0	0.03	0	-	-	-	-	0.03
	72	0	-	-	-	-	0	0.01	0.01
	168	0	0.03	0.01	0.04	-	-	-	0.08
**C_3_ = 1.159 × 10^−5^**	control	0	-	0.02	-	-	-	-	0,02
	6	0.02	0	-	-	-	-	-	0
	12	0	0.02	0	0	-	-	-	0.02
	24	0	0.03	0.01	0	-	-	0	0.04
	48	0	0.01	-	-	-	-	-	0.01
	72	0	0.01	0	-	-	-	-	0.01
	168	0	0	0	-	-	-	-	0

(−) no production; (0) – trace amount; **TAX** (taxifolin), **SILYD** (silydianin), **SILYCHR** (silychristin), **ISOSYLIB A** (isosylibin A), **ISOSYLIB **(isosylibin B), **SILYB A** (silybin A), **SILYB B** (silybin B), **SILYM COMP** (silymarin complex).

**Table 2:** Content of silymarin complex substances (%) and taxifolin (%) in *Silybum marianum* suspensions culture after elicitor treatment.

Compound I (conc. mol/L)	Time of sampling (hours)	TAX (%)	SILYD (%)	SILYCHR (%)	ISOSILYB A (%)	ISOSILYB B (%)	SILYB A (%)	SILYB B (%)	SILYM COMP (%)
**C_1_ = 1.159 × 10^−3^**	control	0.01	0	0.03	-	0	-	0	0.06
	6	0	0.02	-	-	-	-	-	0.02
	12	0	0.02	-	-	-	-	-	0.02
	24	0	0.11	0	-	-	-	-	0.11
	48	0	0.01	0	0.01	-	-	-	0.02
	72	0	0	-	-	0.01	-	-	0.01
	168	0	0.01	-	-	0	-	-	0.02
**C_2_ = 1.159 × 10^−4^**	control	0	-	0.01	-	-	0	-	0.01
	6	0	-	0	-	-	-	-	0
	12	0	-	-	-	0	0.01	-	0.01
	24	0	0	0	-	0	-	-	0
	48	0.01		0	-	-	-	0	0
	72	0	-	0	0	0	-	-	0
	168	0	-	0	-	0	0	0	0
**C_3_ = 1.159 × 10^−5^**	control	0	-	0.01	-	-	-	-	0.01
	6	0	0	-	-	-	-	-	0
	12	0	0.01	-	0	-	-	-	0
	24	0	-	-	-	0	-	-	0.01
	48	0	-	0	-	-	0	0	0
	72	0	-	0	-	-	0	-	0
	168	0	-	0	-	-	-	-	0

(−) no production; (0) – trace amount.

### 2.1. Silybum marianum Callus Culture

The production of silydianin, silychristin, isosilybinin A, isosilybinin B, silybin A, silybin B in callus culture of *S. marianum *after elicitor (compound **I**) treatment was observed. The best elicitation effect in callus culture was found after 12 and 24 h application of the strongest concentration c_1_ (1.159 × 10^−3^ mol/L) of elicitor after which maximum level of silydianin (0.04%) was determined. The maximum content of silymarin complex (0.08%) was induced by 168 h application of c_2_ concentration (1.159 × 10^−4^ mol/L) of elicitor, which represents contents of silydianin (0.03%), silychristin (0.01%) and isosilybin A (0.04%) when compared with control (Table 1). Concentration c_3_ (1.159 × 10^−5^ mol/L) of the elicitor increased taxifolin content (0.01%) after 6 h treatment. The levels of silydianin (0.02%) after 12 h, (0.03%) after 24 h, (0.01%) after 48 h and (0.01%) after 72-hours were reached when c_3_ concentration of elicitor was used (Table 1).

### 2.2. Silybum marianum Suspension Culture

Elicitor at c_1_ concentration increased the content of silydianin at all tested treatment time points: (0.02%) after 6 and 12 h of elicitor application and (0.01%) after 48 and 168 h. The maximum content of silydianin (0.11%) was induced by 24-hours application of c_1 _concentration. The higher levels of other flavonolignans such as isosilybin A (0.01%) after 48 h and isosilybin B (0.01%) after 72 h of c_1_ concentration were determined (Table 2). Elicitor at c_2_ concentration had no noticeable effect on flavonolignan production. This concentration increased only taxifoline content (0.01%) after 48h of duration and silybine A (0.01%) after 12 h of duration. Similar results with c_3_ concentration of elicitor were found (Table 2).

### 2.3. Flavonolignans in Nutrient Medium

The content of flavonolignans was not only determined in the callus and suspension cultures but also in the nutrient media in which *S. marianum* cultures *in vitro* were cultivated. Only taxifolin, silydianin and silychristin were eliminated into the nutrient medium. The highest level of taxifolin in comparison with content in suspension culture (threefold higher) was released into medium. The highest production of taxifolin (0.06%) after application of c_2_ concentration and 72 h duration and (0.05%) after after c_1_ and 24 h duration was determined (Table 3). Sampling at concentration c_2_ after 168 h was not evaluated for contamination of nutrient medium.

**Table 3:** Content of taxifolin, silychristin and silydianin (%) in 100 mL of suspension culture nutrient medium.

Compound I (concentration mol/L)	Time of sampling (hours)	TAXIFOLIN (%)	SILYCHRISTIN (%)	SILYDIANIN (%)
**c_1_ = 1.159** ** × ** **10** **^−^** **^3^**	control	0.02	0	0.01
	6	0.01	0	0.09
	24	0.05	0	0
	48	0.01	0	0.01
	72	0	0	0.21
	168	0.03	0	0
**c_2_ = 1.159** ** ×** ** 10** **^−^** **^4^**	12	0.04	0	0
	24	0	0	0
	72	0.06	0	0
**c_3_ = 1.159** ** × ** **10** **^−^** **^5^**	6	0.04	0	0
	12	0	0	0
	24	0	0	0
	48	0	0	0
	168	0	0	0.05

(0) – trace amount.

The newly synthetized pyrazinecarboxamide derivatives were also tested for their abiotic effects in previous experiments with *Silybum marianum *culture i*n vitro*. The compound 5-*tert*-butyl-*N*-m-tolylpyrazine-2-carboxamide at a concentration 3.71 × 10^−7^ mol/L and within 72 h of elicitation increased flavonolignan production by 893% in suspension culture *versus* control. The flavonolignan production in callus culture after elicitation with *N*-(5-bromo-2-hydroxyphenyl)-5-*tert*-butyl-6-chloropyrazine-2-carboxamide was also increased by about 1039% (24 h elicitation and concentration of 2.59 × 10^−4^ mol/L) [6].

### 2.4. Ononis arvensis Callus Culture

All three tested concentrations of compound **II** increased the flavonoid production in callus culture in a statistically significant manner (Table 4). The best elicitation effect of all elicitor concentrations was at the weakest c_3_ concentration (8.36 × 10^−6^ mol/L) after 168 h. This was a statistically important increase in the flavonoid production (about 1,506%) in comparison with control. Higher flavonoid production was also reached after 24 and 48 h treatment with c_1_ concentration and after 6-and 12 h treatment with c_3_ concentration (Table 4).

**Table 4:** Flavonoid content (%) in callus culture of *Ononis arvensis*.

Compound II (concentration mol/L)	Time of sampling (hours)	Flavonoid content (%)	SD
**c_1_ = 8.36.10^−4^**	control	0.01	0.002
	6	0.025 *	0.004
	12	0.06 *	0.004
	24	0.023 *	0.001
	48	0.057 *	0.002
	72	0.017 *	0.003
	168	0.003	0.001
**c_2_ = 8.36.10^−5^**	control	0.025	0.003
	6	0.079 *	0.007
	12	0.063 *	0.002
	24	0.020	0.002
	48	0.008	0.003
	72	0.006	0.002
	168	0.016	0.003
**c_3_ = 8.36.10^−6^**	control	0.016	0.003
	6	0.218 *	0.005
	12	0.141 *	0.001
	24	0.011	0.004
	48	0.123 *	0.002
	72	0.156 *	0.002
	168	0.241 *	0.003

* statistically significant increase of flavonoid content (*P* ≤ 0.05).

### 2.5. Ononis arvensis Suspension Culture

The maximum content of flavonoids (about 5,800%) was induced by 48 h application of c_3_ concentration. Elicitor concentration c_2_ had no influence on flavonoid production at any time of elicitor treatment (Table 5). Concentration c_2 _on the contrary decreased flavonoid production in the studied times.

**Table 5:** Flavonoid content (%) in suspension culture of *Ononis arvensis*.

Compound II (concentration mol/L)	Time of sampling (hours)	Flavonoid content (%)	SD
**c_1_ = 8.36.10^−4^**	control	0.008	0.002
	6	0.017 *	0.002
	12	0.004	0.003
	24	0.010	0.005
	48	0.210 *	0.003
	72	0.003	0.001
	168	0.002	0.001
**c_2_ = 8.36.10^−5^**	control	0.045	0.005
	6	0.002	0.002
	12	0.006	0.002
	24	0.001	0.001
	48	0.004	0.002
	72	0.020	0.004
	168	0.041	0.003
**c_3_ = 8.36.10^−6^**	control	0.002	0.001
	6	0.044 *	0.004
	12	0.105 *	0.007
	24	0.073 *	0.005
	48	0.118 *	0.006
	72	0.059 *	0.003
	168	0.039 *	0.002

* statistically signifiant increase of flavonoid content (*P *≤ 0.05).

Ring substituted pyrazinecarboxamides were also tested for their abiotic effects in previous studies. These compounds were able to increase the secondary metabolites production in plant cultures *in vitro.* Flavonoid production in *O. arvensis* culture *in vitro *was increased after 6, 12, 72 and 168 h elicitation with 6-chloro-*N*-(4-chloro-3-methylphenyl)-pyrazine-2-carboxamide. The amount of flavonoids released into nutriet medium was not studied.

The maximal flavonoid content (about 900%) was reached after 12 h elicitation with 6-chloro-*N*-(3-iodo-4-methylphenyl)-pyrazine-2-carboxamide. The compound 5-*tert*-butyl-*N*-(4-chloro-3-methyl-phenyl)-pyrazine-2-carboxamide increased the flavonoid content only after 48 and 12 h exposure to this elicitor. The compound 5*-tert*-butyl-6-chloro-*N*-(4-trifluoromethylphenyl)-pyrazine-2-carboxamide increased flavonoid content after 24 h elicitation, while the highest level of flavonoids was reached after 168 h [4].

## 3. Experimental

### 3.1. Plant Material

Callus culture was derived from the germinating seeds of plant *Silybum marianum *(L.) Gaertn. (Asteraceae) and *Ononis arvensis *(L.) (Fabaceae). Seeds for germination were obtained from the Garden of Medicinal Plants, Faculty of Pharmacy in Hradec Kralove. *S. marianum* cultures *in vitro* in the 51st–58th passages were used. Calluses were cultured on Murashige and Skoog medium [12] containing α-naphtylacetic acid (NAA) as growth regulator at a concentration of 5.4 × 10^−5^ mol/L.Callus cultures were cultivated on paper bridges in Erlenmeyer flasks for 35 days and these cultures were incubated in growth chambers at 26 ± 1 °C under a 16 h photoperiod. White light of intensity of 3,500 lux was used. Suspension cultures were cultivated in 250 mL growth flasks with shaking at 80 rpm. Suspensions were kept under the same conditions as callus cultures. The 10th–20th passages were used for our experiment. *Ononis arvensis* culture *in vitro* in the 60th–62nd passages were used. Calluses were cultured on Murashige and Skoog medium [12] containing NAA as growth regulator at a concentration of 5.4 × 10^−5^ mol/L. Callus cultures were cultivated on paper bridges in Erlenmeyer flasks for 28 days and suspension cultures for 21 days. The cultivation of *O. arvensis* cultures *in vitro* was incubated at the same light and temperature conditions according to *S. marianum* culture *in vitro*.

### 3.2. Elicitors

The abiotic elicitors 5-(2-hydroxybenzoyl)-pyrazine-2-carboxamide (compound **I**, Figure 1) at concentrations of 1.159 × 10^−3^ mol/L (c_1_); 1.159 × 10^−4^ mol/L (c_2_); 1.159 × 10^−5^ mol/L (c_3_) and *N*-(2-bromo-3-methylphenyl)-5-*tert*-butylpyrazin-2-carboxamide (compound **II**, Figure 1) at concentrations of 8.36 × 10^−4^ mol/L (c_1_); 8.36 × 10^−5^ mol/L (c_2_); 8.36 × 10^−6^ mol/L (c_3_) were tested. Callus and suspension cultures of *O. arvensis* were elicited only by compound **II** and callus and suspension cultures of *S. marianum* were elicited only by compound **I**. The elicitor (compound **I**) was added to the callus culture on the 30th day of cultivation and to the suspension culture on the 21st day of cultivation of *S. marianum.* The elicitor (compound **II**) was added to the callus culture on the 28th day of cultivation and to the suspension culture on the 21st day of cultivation of *O. arvensis*. Six; 12; 24; 48; 72 and 168 h after elicitor application, the callus and cells from suspension cultures were sampled, dried and the content of secondary metabolites (flavonolignans and flavonoids) was determined. Simultaneously, the controls (without elicitors) were run for 24 and 168 h.

### 3.3. Analysis of Flavonolignans and Flavonoids

The content of flavonolignans in *S. marianum* cultures *in vitro* was determined by HPLC on a UNICAM CRYSTAL 200 Liquid Chromatograph, using a LiChrospher RP-18 (250 mm × 4 mm) column according Czech Pharmacopeia 2009 [13]. The content of flavonoids in *O. arvensis* cultures *in vitro *was evaluated on a spectrophotometer CECIL 1000 SERIES according Czech Pharmacopeia 2009 [14]. All experimental analyses were carried out in a minimum of three independent samples for each elicitation period and each concentration of elicitor Statistical significance was calculated using ANOVAtest for unpaired data (*P* ≤ 0.05).

## 4. Conclusions

The results of the performed experiments show that successful elicitation is subject to many factors that are specific to each elicitor and for each explant culture. An important part of successful elicitation is the type of elicitor, its concentration and the time of its action. All results clearly indicate that the tested compound 5-(2-hydroxybenzoyl)-pyrazine-2-carboxamide (**I**) has shown to be an effective elicitor of flavonolignans and taxifolin production in *Silybum marianum culture in vitro*. The compound *N*-(2-bromo-3-methylphenyl)-5-*tert*-butylpyrazine-2-carboxamide (**II**) also seems to be an effective elicitor for flavonoid production in *Ononis arvensis *culture *in vitro*. These newly synthesized chemical compounds were proven to be promising elicitors for the induction of secondary metabolism in explant cultures.
